# The Significance of the Regulation Between tRF‐31‐U5YKFN8DYDZDD and BMPER in the Pathogenesis of Gastric Cancer (GC)

**DOI:** 10.1111/jcmm.71320

**Published:** 2026-08-14

**Authors:** Yuejiao Huang, Xun Li, Yu Zhang, Xinliang Gu, Shiyi Qin, Ming Zheng, Han Wang, Shaoqing Ju

**Affiliations:** ^1^ Department of Hematology and Oncology Affiliated Hospital of Nantong University, Medical School of Nantong University Nantong Jiangsu China; ^2^ Medical School of Nantong University Nantong University Nantong Jiangsu China; ^3^ Department of Laboratory Medicine Affiliated Hospital of Nantong University Nantong Jiangsu China; ^4^ Department of General Surgery Affiliated Hospital of Nantong University Nantong Jiangsu China

**Keywords:** BMPER, gastric cancer, tRF‐31‐U5YKFN8DYDZDD, tRNA‐derived small RNAs

## Abstract

tsRNAs are a kind of small non‐coding single‐stranded RNA, which play an important role in many kinds of tumours. Previous studies have identified that tRF‐31‐U5YKFN8DYDZDD can be used as a novel tumour biomarker for the diagnosis and prognosis of gastric cancer (GC). In this study, we further explored the regulatory effect of tRF‐31‐U5YKFN8DYDZDD on tumour biological function in GC cells and related regulatory mechanisms. Inhibition of tRF‐31‐U5YKFN8DYDZDD can inhibit the proliferation, invasion, migration and angiogenesis of GC cells, while overexpression of tRF‐31‐U5YKFN8DYDZDD has the opposite effect. BMPER was shown to be a direct target of tRF‐31‐U5YKFN8DYDZDD in GC. Furthermore, the cytological effects of stable overexpression of BMPER were similar to the inhibition of tRF‐31‐U5YKFN8DYDZDD. And the attenuation of BMPER expression rescued the tRF‐mediated promotion of GC cells. WB showed that up‐regulation of tRF‐31‐U5YKFN8DYDZDD could increase the phosphorylation level of ERK and Smad1/5, and promote the expression of epithelial mesenchymal transition (EMT) and matrix metalloproteinases. Animal experiments in vivo show that down‐regulation of tRF‐31‐U5YKFN8DYDZDD can effectively inhibit tumour growth. These data suggest that tRF‐31‐U5YKFN8DYDZDD is a new tumour‐promoting factor and may be a potential new therapeutic target for GC.

**Abbreviations**
Ago2: argonaute 2
Ang: angiogenin
CAM: Chicken chorioallantoic membrane
DAB: diaminobenzidine
ECL: Electrochemiluminescence
EMT: epithelial mesenchymal transition
FDA: Food and Drug Administration
GC: Gastric cancer
hATTR: hereditary thyroxine‐mediated amyloidosis
HUVECs: Human umbilical vein endothelial cells
IHC: Immunohistochemistry
i‐tRF: endogenous tRF
MUT: mutant vectors
MVD: microvessel density
ncRNAs: non‐coding RNA
OD: absorbance values
PBS: phosphate‐buffered saline
pDC: plasma‐like dendritic cells
PI: propidium iodide
PVDF: polyvinylidene fluoride
RIP: RNA binding protein immunoprecipitation
SDS‐PAGE: sodium dodecyl sulfate‐polyacrylamide gel electrophoresis
TBST: Tris‐buffered saline with Tween 20
TCGA: the cancer genome atlas
Tcm: central memory T cell
Tem: effector memory T cell
tiRNAs: tRNA halves
tRFs: tRNA‐derived fragments
tsRNAs: transfer RNA‐derived fragments
WT: wild‐type

## 1 Introduction

Although the incidence and mortality of gastric cancer (GC) have gradually shown a decreasing trend in recent years with the continuous improvement of physical examination and treatment [1, 2]. In recent decades, a group of non‐coding RNA (ncRNAs) related to cancer biology is transfer RNA‐derived small RNAs (tsRNAs). It is important regulators that activate or influence the signalling pathways during the occurrence and development of cancer [3, 4]. At present, the exact role of tsRNAs in GC is still at the initial stage. The research on the expression and mechanism of tsRNAs in GC will greatly promote the progress of diagnosis and treatment.

tsRNAs play different roles in different types of cancers. Lee et al. reported that a tRF‐1, tRF‐1001, derived from the precursor tRNA^Ser^, is highly expressed in several cancer cell lines and is an essential regulator for the proliferation of prostate cancer [5]. Honda et al. announced a new tiRNA for the first time, which is a sex hormone‐dependent tiRNA and can promote the cell proliferation of breast cancer and prostate cancer [6]. tRF‐03357 promotes cell proliferation, migration and invasive ability in high‐grade serous ovarian cancer [7]. tRF‐Leu‐CAG has been shown to promote cell proliferation and cell cycle regulation in non‐small cell lung cancer [8]. tiRNA‐Gly promotes the migration and proliferation of papillary thyroid cancer cells by binding to RBM17 and inducing alternative splicing [9]. tRF‐Lys‐CTT‐010, on the other hand, promotes the malignant progression of triple‐negative breast cancer by regulating glucose metabolism through inhibition of G6PC [10]. Of course, in addition to studies in solid tumours, studies have also been involved in hematologic tumours, especially in chronic lymphocytic leukaemia [11, 12]. With the gradual deepening of mechanism research, it has been found that the regulation of tsRNA in tumours involves a variety of signal pathways. For example, 5′‐tiRNA‐Val was shown to inhibit the Wnt/β‐catenin signalling pathway by targeting the 3′‐UTR region of FZD3 mRNA in breast cancer cells [13]. tRF/miR‐1280 inhibits the colon carcinogenesis by activating Notch signalling pathway [14]. With the popularization of the research, it is believed that the expression and mechanism of tsRNAs in tumours will be fully explored.

In contrast, tRF‐5026a, which can also be referred to as tRF‐18‐79MP9P04, was found to exert tumour suppressive effects through the PTEN/PI3K/AKT signalling pathway and is a promising therapeutic target for GC [15]. tRF‐24‐V29K9UV3IU may inhibit the progression of GC by inhibiting the proliferation, migration and invasion of tumour cells, while promoting apoptosis by regulating the Wnt signalling pathway [16]. Based on the previous study, we screened tRF‐31‐U5YKFN8DYDZD by RNA high‐throughput sequencing, which was differentially expressed in GC and paraneoplastic tissues. We also study its role in tumorigenesis and development in GC and analyse its potential clinical application value.

## 2 Materials and Methods

### 2.1 Tissue Specimens

All sampling requirements, diagnostic criteria, informed consent and ethical requirements were the same as those of the previous paper [17].

### 2.2 Cell Culture and Transfection

Cell culture was performed as per our previously described procedures [17]. Human umbilical vein endothelial cells (HUVECs) were grown in DMEM, supplemented with 10% FBS (Gibco, New York, USA). The cells were granted from Tongji University (Shanghai, China). Prior to conducting the experiments, the cell lines were rigorously identified by short tandem repeat and tested to confirm their absence of contamination, including scrutiny for mycoplasma. Cell transfection was undertaken with Lipofectamine 3000 according to the manufacturer's instructions (Invitrogen, California, USA). BMPER sequences were synthesized and inserted into pHBLV vector (GeneChem, Shanghai, China). The RNA isolation and qRT‐PCR assays were performed as described previously [17].

### 2.3 CCK‐8 Assay

GC cells (5 × 104/well) were seeded in 96‐well plates in 100 μL after transfection. 10 μL CCK‐8 reagent (Dojindo Molecular Technologies, Kumamoto, Japan) was added to each well and incubated for 2 h at 37°C in a CO2 incubator. And 450 nm absorbance values (OD) were measured at 12, 24, 48, 72 and 96 h.

### 2.4 Colony Formation Assay

After digestion and collection, the fine density of the transfected cells was adjusted to 1 × 10^3^ cells per well. The culture liquid was changed every 4 days until 2 weeks. Then the cells were fixed with 4% paraformaldehyde (Beyotime, ShangHai, China) for 30 min and stained with crystal violet (Beyotime, ShangHai, China) for 10 min.

### 2.5 EdU

300 μL/well (1 × 10^5^ cells/mL) of cell suspension was added to the 24‐well plate and incubated. After the cells adhered to the wall, the culture medium was replaced with the same volume of complete culture medium containing 50 μM EdU, and the incubation continued for 2 h. Cells were fixed with 4% paraformaldehyde and permeabilized with Triton X‐100 for 15 min each. The Click reaction solution was configured according to the sequence and volume. Add 100 μL/well of the Click reaction solution and shake gently in the dark for 30 min. 200 μL/well Hoechest 33,342 staining solution was added to avoid light and gently vibrate for 10 min.

### 2.6 Transwell Assay

Transfected cells were collected, resuspended with 150 mL basal medium and inoculated into the upper chamber of Transwell (Corning, New York, USA), and 600 mL of 20% complete medium was added to the lower chamber. Similarly, cells were resuspended on Transwell chambers pre‐layered with matrix gel (Corning, New York, USA), and the number of invading cells was detected after 48 h of incubation for invasion. At the end of the culture, the cells on the upper membrane surface were gently wiped off with a cotton swab. The cells on the lower membrane surface were fixed with 4% paraformaldehyde and placed in 0.1% crystal violet for 15 min.

### 2.7 Wound Healing

When the cells reached 100% confluence, the cell monolayers were mechanically disrupted using a sterile 100 μL pipette tip to generate a linear wound. Plates were washed twice with PBS to remove the suspended cells. Images of the cells were captured at 0 and 24 h and the average distance migrated by the cells was measured under a microscope (Olympus, Tokyo, Japan).

### 2.8 Dual‐Luciferase Reporter Assay

The constructed BMPER wild‐type (WT) and mutant vectors (MUT) were co‐transfected with tRF‐31‐U5YKFN8DYDZD mimics (Ribobio, GuangZhou, China) into GC cell lines. After 48 h of transfection, the cells were treated according to the manufacturer's instructions (Promega, Wisconsin, USA), and the Firefly and Renilla luciferase (hRluc) activities were detected by spectrophotometer. The levels of hRluc were normalized and calculated to firefly luciferase levels.

### 2.9 RNA Binding Protein Immunoprecipitation (RIP)

RIP assays were performed by using the RNA Immunoprecipitation Kit (BersinBio, Guangzhou, China) according to the protocol. Briefly, 10^7^ cells were lysed using RIP lysis buffer with protease and RNase inhibitors. The cell lysates were incubated with 5 mg of nonspecific mouse IgG or anti‐argonaute 2 (Ago2) antibody (Cat No. 67934–1‐Ig, Proteintech, Illinois, USA) coated magnetic beads with rotation at 4°C overnight, respectively. The immunoprecipitate was digested with proteinase K, and the immunoprecipitated RNAs were subjected to qRT‐PCR and gel‐staining analyses to detect tRF‐31‐U5YKFN8DYDZDD and BMPER enrichment.

### 2.10 Apoptosis Assay

The transfected cells were collected and resuscitated with the binding buffer (Vazyme, Nanjing, China). According to the manufacturer's protocol, 5 μL Annexin V‐FITC solution and 10 μL propidium iodide (PI) were added in turn and incubated on ice for 15 min, protected from light. With the help of laboratory technicians in the Clinical Centre of the Affiliated Hospital of Nantong University, flow cytometry (FACScan, BD Biosciences, New Jersey, USA) was used to detect apoptosis within 30 min.

### 2.11 HUVECs Tube Formation

First, 100 μL Matrigel (Corning, New York, USA) was plated in each well of a precooled 24‐well plate after thawing at 4°C and allowed to polymerize at 37°C in the incubator for 30 min. At the same time, the tumour‐conditioned medium was collected from the supernatant of different transfected GC cells mixed with fresh medium (1:1). Then, the HUVECs were mixed with 300 μL of the conditioned medium at a concentration of 1 × 10^4^ cells/mL and seeded into the precoated 24‐well plates, followed by cultivation at 37°C. Tube formation by HUVECs was quantitatively assessed using the Angiogenesis Analyser plugin for ImageJ.

### 2.12 Chicken Chorioallantoic Membrane (CAM)

Briefly, the embryonic chicken eggs were incubated in a humidified atmosphere of 70% at 37°C for 1 week, and their CAM was exposed to observe angiogenesis. On embryonic day 8, an artificial air chamber was created in the avascular zone to separate the shell membrane and the chorioallantoic membrane, and then a 0.2 × 0.2 cm window on the shell membrane. After that, 40 μL of 1 × 10^6^ transfected cell suspension was dropped into a gelatin sponge and added onto the CAM or 200 μL of tumour‐conditional medium obtained from transfected GC cells was injected through the small puncture window.

### 2.13 Western Blot (WB)

Protein extraction and electrophoresis assays were performed as described in previous studies [18]. Then the protein bands were displayed with Electrochemiluminescence (ECL) reagent (Epizyme, Shanghai, China). The antibodies used in this study were: rabbit anti‐BMPER (Immunoway, California USA); rabbit anti‐Vimentin, E‐cadherin, N‐cadherin, ERK, p‐ERK, Bim and PTEN (Cell Signalling Technology, Massachusetts, USA); mouse anti‐Bcl‐2 (Cell Signalling Technology, Massachusetts, USA); rabbit anti‐MMP2 and MMP9 (Proteintech, Illinois, USA); rabbit anti‐Bax (BBI Solutions, Cardiff, UK); mouse anti‐GAPDH antibody (ABclonal, Wuhan, China).

### 2.14 Animal Study

BALB/c nude mice (4 weeks old) were purchased from Nantong University Animal Center and housed in pathogen‐free conditions. The MKN‐45 cells was transfected with tRF‐31‐U5YKFN8DYDZDD long‐acting inhibitory vector (antagomir) and interference control (NC), respectively. The cells of the above two groups and the untransfected blank control group (Mock) were injected into the subaxillary subcutaneous of nude mice to establish the tumour transplantation model (5 × 10^6^ cells in 60 μL PBS + 60 μL Matrigel). Subcutaneous tumour formation was observed 1 week after injection, and then the tRF‐antagomir and NC were each randomly divided into two groups. One group was selected for combination chemotherapy, i.e., 5‐Fu was injected intraperitoneally every three days. The mice were sacrificed 30 days after injection.

### 2.15 Immunohistochemistry (IHC)

The tissues were fixed, dehydrated, transparent, paraffin‐embedded and sliced. Then the sections were dewaxed, hydrated and repaired with antigen for immunohistochemical staining. The primary antibody was incubated overnight at 4°C and the secondary antibody was incubated for 1 h at room temperature. Finally, diaminobenzidine reagent coloration and haematoxylin staining were applied. These above operations were automated with the help of automatic dewatering, embedding, dyeing and sealing machine in the Department of Pathology, Affiliated Nantong Third Hospital of Nantong University.

### 2.16 Statistical Analysis

All data were analysed by SPSS version 20.0 (IBM SPSS Statistics, Chicago, USA), and GraphPad Prism v8.0 (The GraphPad Software, La Jolla, California, USA). Each experiment was independently repeated at least three times. The data are expressed as the mean value ± standard deviation (SD). Two‐tailed unpaired *t*‐test was adopted for the comparison of two independent samples, while one‐way analysis of variance (ANOVA) was used to compare multiple independent samples. *p < 0.05; **p < 0.01; ***p < 0.001; ****p < 0.0001 was considered statistically significant.

## 3 Results

### 3.1 tRF‐31‐U5YKFN8DYDZDD Affects the Proliferation, Invasion and Migration Ability of GC Cells

**FIGURE 1:**
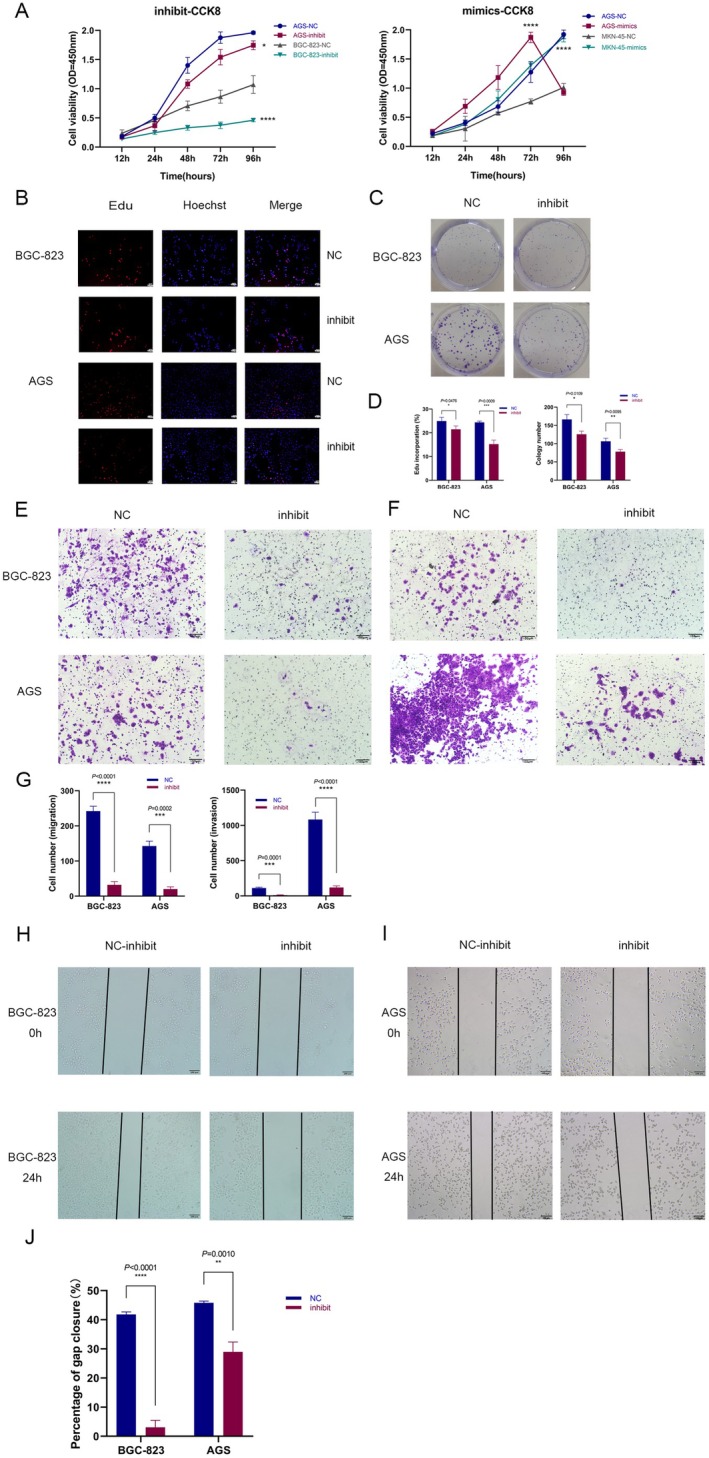
Effect of interfering tRF‐31‐U5YKFN8DYDZDD on the biological ability of GC cells. The proliferation, migration and invasion of GC cells after interference with tRF‐31‐U5YKFN8DYDZDD by CCK‐8 (A), EdU (B, D left), clone formation (C, D right), migration (E, G left), invasion (F, G right) and wound healing (H–J) assay.

Firstly, the interference and overexpression efficiency of tRF‐31‐U5YKFN8DYDZDD were measured by qRT‐PCR (Figure S1). Next, the CCK‐8, EdU and colony formation assay results showed that cell proliferation was diminished after interfering with tRF‐31‐U5YKFN8DYDZDD (Figure 1A–D). Among them, due to the rapid growth of AGS cells, the control group died at 96 h due to overgrowth and lack of nutrition, and the OD value decreased sharply. Next, the transwell (Figure 1E,G left) and wound healing (Figure 1H–J) assays also revealed that migration was significantly suppressed in tRF‐31‐U5YKFN8DYDZDD knockdown cell lines. Similarly, it was found that the number of cell invasions decreased after interfering with the expression of tRF‐31‐U5YKFN8DYDZDD (Figure 1F,G right). On the contrary, after transfection of mimics of tRF‐31‐U5YKFN8DYDZDD, the opposite conclusion was obtained by repeating the above experiments, that is, the overexpression of tRF‐31‐U5YKFN8DYDZDD can promote the proliferation, migration and invasion of GC cells (Figure S2A–F).

### 3.2 Validation of the Combination of tRF‐31‐U5YKFN8DYDZDD and BMPER

**FIGURE 2:**
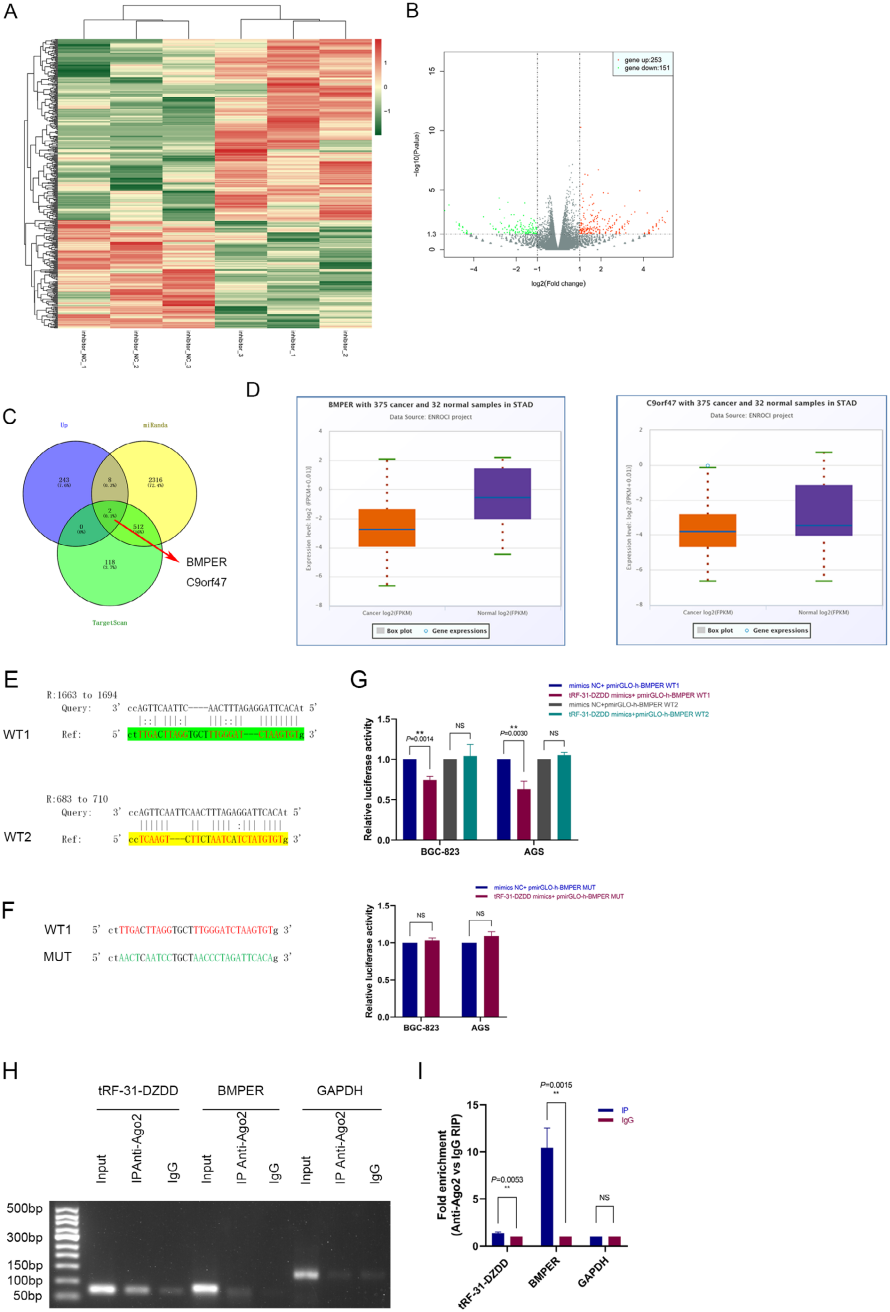
Predictive analyses of tRF‐31‐U5YKFN8DYDZDD target genes. The clustered heatmap (A) and volcano plots (B) showed the differentially expressed mRNAs in 3 pairs of NC‐inhibit and tRF‐31‐U5YKFN8DYDZDD inhibit cells. (C) Venn diagram evaluated the overlapped genes between miRandan, TargetScan and high‐throughput sequencing predictions. (D) Prediction of BMPER and C9orf47 expression in the TCGA database by ENCORI analysis. Schematic diagram of the combination (E) and mutant construction (F) of tRF‐31‐U5YKFN8DYDZDD to 3′UTR of BMPER. (G) Dual luciferase reporter assay to detect complementary binding of tRF‐31‐U5YKFN8DYDZDD to BMPER. (H) The qRT‐PCR product of RIP assays was run on 2% agarose gel. (I) RIP assays were performed on extracts from GC cells using an antibody against Ago2.

In order to further clarify the changes of gene expression regulated by tRF‐31‐U5YKFN8DYDZDD, NC‐inhibit and tRF‐31‐U5YKFN8DYDZDD inhibit were transfected into AGS for high‐throughput sequencing. The results showed that a total of 404 genes were differentially expressed, among which 253 genes were up‐regulated (Figure 2A,B). Venn diagram of the up‐regulated genes with the target genes predicted by miRanda and TargetScan showed that both BMPER and C9orf47 were the most potential target genes (Figure 2C). The expression of BMPER and C9orf47 in GC and normal gastric tissue in the cancer genome atlas (TCGA) database was analysed by ENCORI (https://starbase.sysu.edu.cn/panCancer.php). BMPER was low in GC, while C9orf47 showed no significant difference between the two groups (Figure 2D). It is suggested that BMPER is a potential downstream regulatory target gene of tRF‐31‐U5YKFN8DYDZDD.

Through online database analysis, we predicted that there may be two main binding sites between tRF‐31‐U5YKFN8DYDZDD and BMPER (Figure 2E), and constructed BMPER WT vectors 1 and 2, respectively. Dual luciferase reporter gene assays showed that the fluorescence signal of hRluc decreased in the cells co‐transfected with tRF‐31‐U5YKFN8DYDZDD mimics and BMPER WT1 vector (Figure 2G upon). Then, we constructed BMPER MUT vectors 1 according to the binding sites (Figure 2F). The results showed that the fluorescence signals were not reduced in cells both co‐transfected with MUT1 (*p* > 0.05, Figure 2G below). It is suggested that there are binding sites between tRF‐31‐U5YKFN8DYDZDD and BMPER, and the binding sites are in the range of 1663–1694. Subsequently, RIP assays were also performed in AGS cells with antibodies against Ago2, and the results showed that tRF‐31‐U5YKFN8DYDZDD and BMPER were obviously enriched (Figure 2H,I).

**FIGURE 3:**
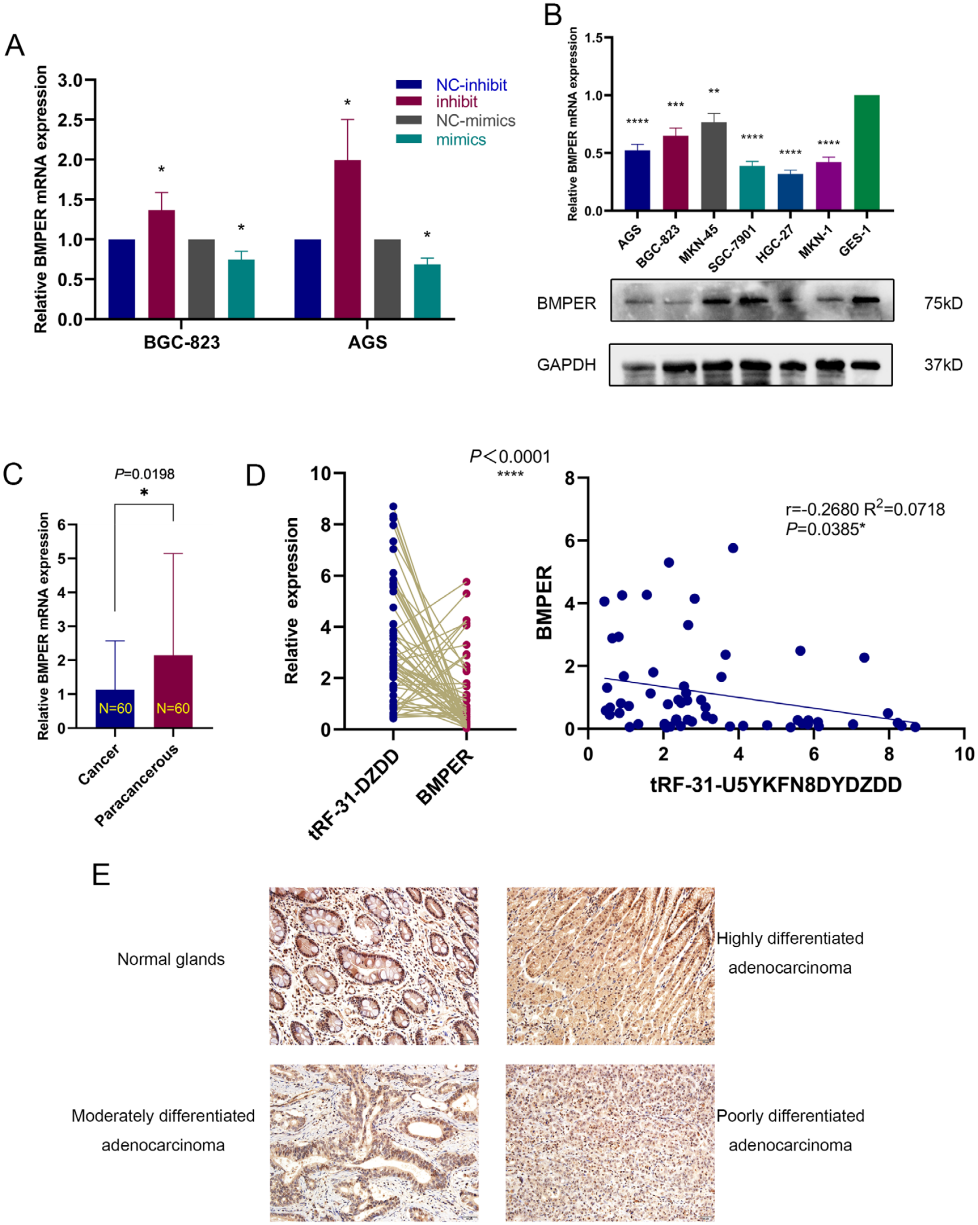
Expression of BMPER in cell lines and tissues of GC. (A) The expression of BMPER was detected by qRT‐PCR after interference or overexpression of tRF‐31‐U5YKFN8DYDZDD. (B) The expression of BMPER in GC cell line. (C) The differential expression of BMPER between GC and adjacent tissue. (D) Correlation between expression of tRF‐31‐U5YKFN8DYDZDD and BMPER in GC. (E) IHC assays show the expression of BMPER in tissue.

The qRT‐PCR results showed that overexpression of tRF‐31‐U5YKFN8DYDZDD could down‐regulate the mRNA expression of BMPER, while interference with tRF‐31‐U5YKFN8DYDZDD could up‐regulate the mRNA expression of BMPER (Figure 3A). Next, we found that the expression of BMPER in GC cell lines was lower than that in normal gastric mucosal epithelial cells GES‐1 (Figure 3B). It is worth noting that the mRNA level of BMPER in 60 pairs GC tissues was significantly lower than that in paracancerous specimens (Figure 3C). Meanwhile, the expression of tRF‐31‐U5YKFN8DYDZDD showed an opposite trend to that of BMPER (Figure 3D). The IHC assays also demonstrated the low expression of BMPER in the adenocarcinoma regardless of the degree of differentiation (Figure 3E). It was also found that the low expression of BMPER was significantly associated with lymph node metastasis, higher TNM stage, nerve invasion and vascular invasion (Table 1), but not with age, sex, pathological differentiation, tumour size, etc.

**TABLE 1:** Association between BMPER mRNA expression and clinicopathologic parameters in 60 GC specimens.

Parameters	Total	BMPER expression		*p*
		Low (<median)	High (≥median)	
Age				0.425
< 60	21	11	10	
≥ 60	39	18	21	
Gender				0.575
Male	21	10	11	
Female	39	19	20	
Differentiation grade				0.464
Well‐moderate	40	20	20	
Poor‐undifferentiation	20	9	11	
Tumour size				0.514
< 5 cm	34	16	18	
≥ 5 cm	26	13	13	
Depth of invasion				0.051
T1–T2	24	8	16	
T3–T4	36	21	15	
Lymph node metastasis				0.000***
Negative	25	5	20	
Positive	35	24	11	
TNM stage				0.000***
I–II	29	7	22	
III–IV	31	22	9	
Lauren classification				0.437
Intestinal type	17	7	10	
Diffuse type	20	12	8	
Mixed type	23	10	13	
Nerve invasion				0.020*
Negative	34	12	22	
Positive	26	17	9	
Vascular invasion				0.019*
Negative	19	5	14	
Positive	41	24	17	
C‐erbB‐2				0.632
Negative	54	26	28	
Positive	6	3	3	
CEA				0.289
Negative (< 5 ng/mL)	26	11	15	
Positive(≥ 5 ng/ml)	34	18	16	
CA199				0.102
Negative (< 37 U/mL)	33	13	20	
Positive(≥ 37 U/ml)	27	16	11	
CA724				0.063
Negative (< 10 U/mL)	34	13	21	
Positive(≥ 10 U/ml)	26	16	10	

*Note:* Statistical analyses were performed by the Pearson *χ* ^2^ test. *p < 0.05 and ***p < 0.001 was considered significant.

### 3.3 Effect of tRF‐31‐U5YKFN8DYDZDD Regulating BMPER on the Function of GC Cells

**FIGURE 4:**
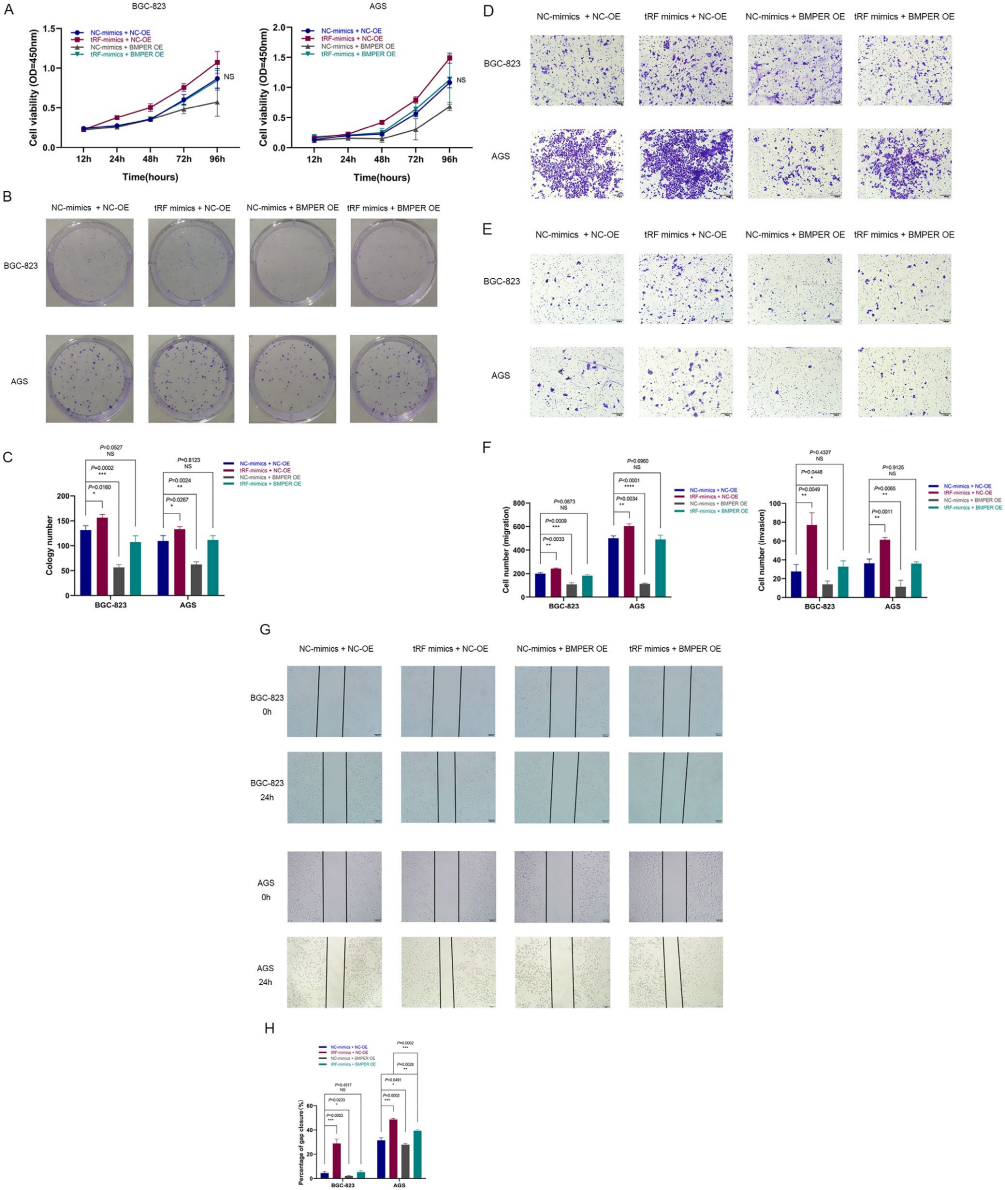
Effect of tRF‐31‐U5YKFN8DYDZDD regulating BMPER on the biological ability of GC cells. The proliferation after co‐transfection with tRF‐31‐U5YKFN8DYDZDD mimics in BMPER stable transfer GC cells by CCK‐8 (A) and clone formation (B, C) assays. Transwell (D–F) and wound healing (G, H) assays were used to detect the migration and invasion of GC cells after co‐transfection of tRF‐31‐U5YKFN8DYDZDD mimics with overexpressed BMPER.

The expression overexpression efficiency was verified at the mRNA level and protein level in BMPER stably transfected cell lines (Figure S3A,B). CCK‐8, clonogenic, transwell and wound healing assay showed that after stable overexpression of BMPER, the proliferation, invasion and migration of GC cells were diminished (Figure S4A–G). The results of CCK‐8 and cell cloning experiments showed that the cell proliferation ability was low in BMPER overexpression stable beads, while transfection of BMPER overexpression could partially reverse the inhibitory effect of tRF‐31‐U5YKFN8DYDZDD mimics (Figure 4A–C). Similarly, the ability of invasion and migration of cells co‐transfected with tRF‐31‐U5YKFN8DYDZDD mimics in BMPER overexpression group was significantly higher than that in BMPER overexpression group (Figure 4D–H).

### 3.4 Effect of tRF‐31‐U5YKFN8DYDZDD‐BMPER Regulation on Microangiogenesis

**FIGURE 5:**
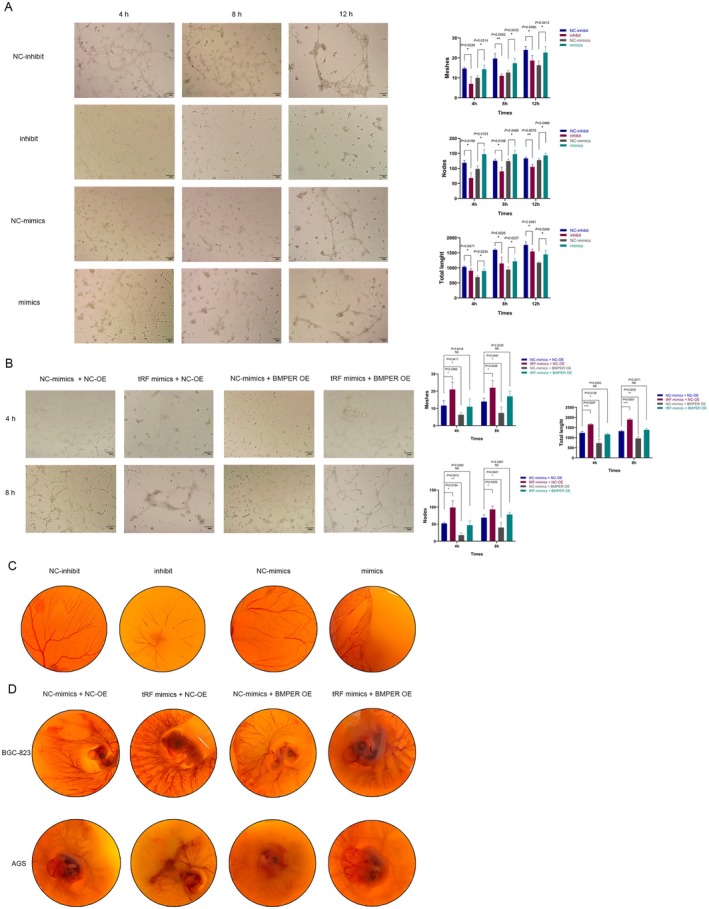
Effect of tRF‐31‐U5YKFN8D3YDZDD regulating BMPER on the microangiogenesis. (A,B) Tube formation of HUVEC cells treated with control medium or conditioned medium obtained from GC cells. The meshes, nodes and the total tube length were determined by Image J. (C) The CAM assay was performed using embryonated chicken eggs treated with the control medium or conditioned medium obtained from GC cells. (D) Representative images of the CAM assay treated with single‐transduced or co‐transfected GC cells.

BMPER is clearly involved in the regulation of angiogenesis [19, 20], which is a critical and rate‐limiting step in tumour growth and metastasis, though not the sole necessary condition. The results showed that the tube‐forming ability of HUVECs was significantly weaker in the conditioned medium transfected with tRF‐31‐U5YKFN8DYDZDD interference than that of the control group at 4 h, 8 h and 12 h, respectively (Figure 5A above). On the contrary, the conditioned medium formed after overexpression of tRF‐31‐U5YKFN8DYDZDD significantly promoted the tube formation ability (Figure 5A below). In the CAM experiment, it was observed that the vascular network became thinner and even partially dissolved, while the conditioned medium transfected with tRF‐31‐U5YKFN8DYDZDD mimics became denser (Figure 5C). In response, the co‐transfected cells were directly added to the absorbable gelatin sponge and incubated on the surface of CAM. Compared with the injection of the conditioned medium, the continuous surface incubation of tumour cells has a more obvious effect on neovascularization. Moreover, BMPER could partially reverse the promotion effect of tRF‐31‐U5YKFN8DYDZDD overexpression on angiogenesis (Figure 5B,D).

### 3.5 tRF‐31‐U5YKFN8DYDZDD‐BMPER Affects the Malignant Progression of GC Through ERK and Smad Signalling Pathways

**FIGURE 6:**
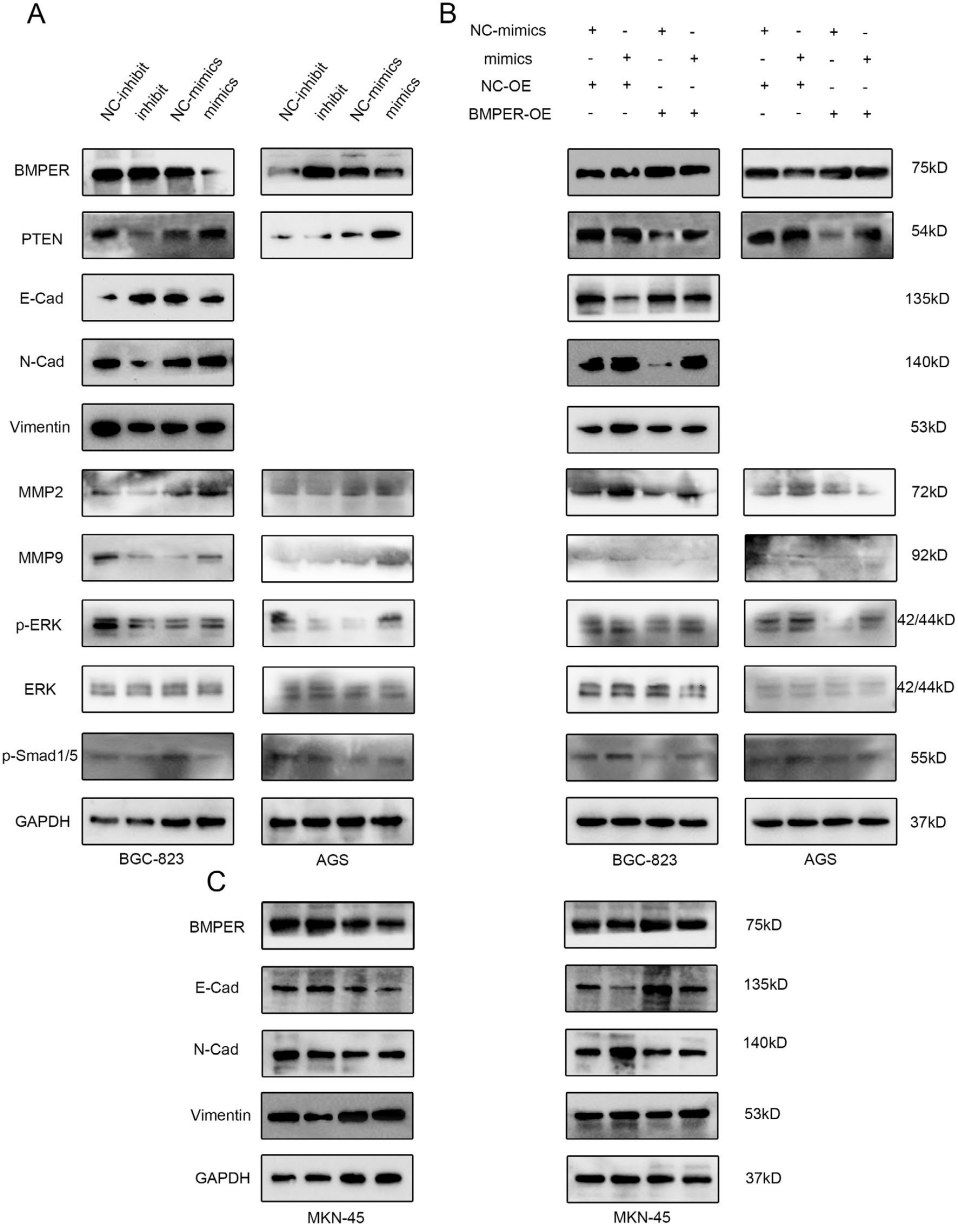
Effect of tRF‐31‐U5YKFN8DYDZDD‐BMPER regulation on phosphorylation of ERK and Smad1/5. (A, B) WB was used to detect the expressions of proliferation, EMT, angiogenesis, apoptosis markers and ERK and Smad signalling pathways in BGC‐823 and AGC cells. (C) The protein levels of EMT‐associated proteins in MKN‐45 cells were determined using WB. GAPDH was used as the internal control.

Previous studies have suggested that BMPER activates ERK and Smad signalling pathways by regulating the expression of the BMP family [21, 22], and the activation of the two is also a classical pathway involved in the regulation of angiogenesis and epithelial mesenchymal transition (EMT). The results of WB experiments first verified that the protein expression of BMPER was significantly increased after interference with tRF‐31‐U5YKFN8DYDZDD, and after overexpression of tRF‐31‐U5YKFN8DYDZDD, BMPER was significantly increased. At the same time, after interfering with tRF‐31‐U5YKFN8DYDZDD, the expression of PTEN, N‐cad, Vimentin, MMP2 and MMP9 decreased, while E‐cad increased, which is consistent with the regulation of proliferation, migration, invasion and angiogenesis of GC cells (Figure 6A,C left). Furthermore, the expression of tRF‐31‐U5YKFN8DYDZDD is positively correlated with the phosphorylation levels of ERK and Smad1/5 (Figure 6A). The above‐mentioned expression changes are also verified in the recovery experiment (Figure 6B). Due to the absence of EMT phenotypic expression in the AGS cell line, the regulation of tRF‐31‐U5YKFN8DYDZDD on E‐cad, N‐cad and Vimentin in another GC cell line MKN‐45 was confirmed (Figure 6C).

### 3.6 tRF‐31‐U5YKFN8DYDZDD Interference Can Inhibit the Growth of GC in Vivo

**FIGURE 7:**
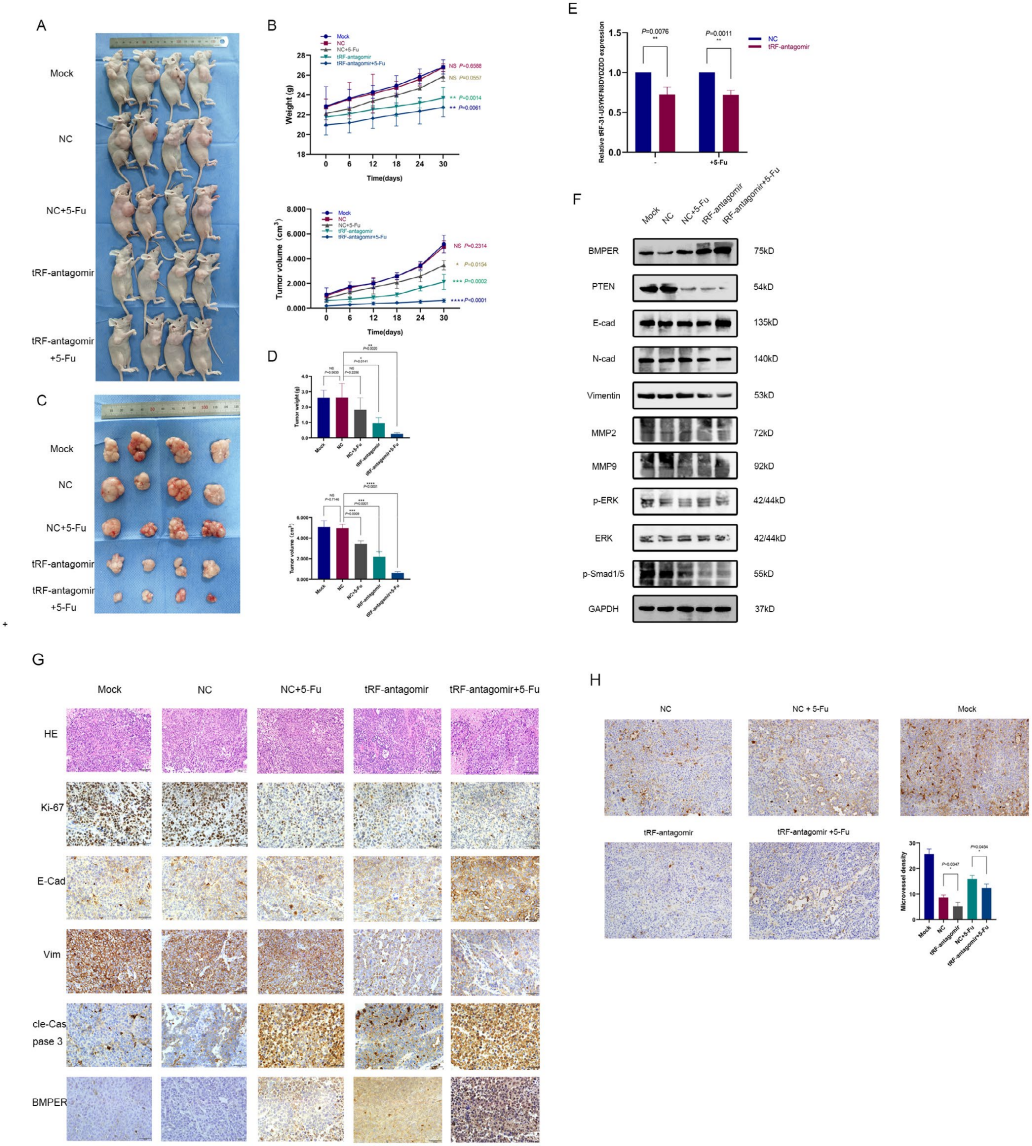
Inhibiting the expression of tRF‐31‐U5YKFN8DYDZDD inhibits tumour growth in vivo. (A) Grouping and tumour formation of nude mice. (B) Growth curve of overall weight and tumour volume in nude mice. (C) Overall diagram of tumour exfoliation. (D) Statistical chart of tumour weight and volume. (E) The levels of tRF‐31‐U5YKFN8DYDZDD were measured by qRT‐PCR in vivo. (F) The levels of protein of BMPER and other markers were measured by WB in vivo. (G) HE staining and the expression of Ki‐67, cleaved‐Caspase 3, E‐cadherin, Vimentin and BMPER in GC subcutaneous tumour tissues by IHC. Original magnification × 400. (H) CD34 staining (20 ×) and MVD in vivo.

Clinical data and experiments in vitro have confirmed that inhibiting the expression of tRF‐31‐U5YKFN8DYDZDD can slow down the proliferation. It was observed that the volume and weight of the subcutaneous tumour in the tRF‐31‐U5YKFN8DYDZDD‐antagomir group were significantly smaller than those in NC and Mock (Figure 7A–D). The results showed that the tumour suppressive effect of tRF‐31‐U5YKFN8DYDZDD‐antagomir is comparable to that of conventional chemotherapeutic drug 5‐Fu, and the combination therapy has a more significant anti‐tumour effect (Figure 7A–D).

qRT‐PCR verified that the expression of tRF‐31‐U5YKFN8DYDZDD in the tRF‐antagomir group was lower than that in NC and Mock (Figure 7E). IHC staining showed that Ki‐67 decreased in the tRF‐antagomir and 5‐Fu groups, and the lowest expression was found in the tRF‐antagomir+5‐Fu group, while cleaved‐Caspase 3 increased significantly in both 5‐Fu groups, but in the tRF‐antagomir group was lower than that two. Compared with the control group, the expression of E‐cad in the tRF‐antagomir group increased and that of Vimentin decreased. Meanwhile, BMPER was localized in the cytoplasm, with a significant augmentation observed in the tRF‐antagomir group, reaching its zenith in the tRF‐antagomir+5‐Fu cohort (Figure 7G). In addition, we repeatedly detected the expression of the markers by WB in vivo, and the results were consistent (Figure 7F). It is also shown that the apoptosis rate of tRF‐31‐U5YKFN8DYDZDD interference was slightly increased, but there was no statistical difference (Figure S5A–H). Furthermore, there is no variation tendency between apoptosis‐related Bax, Bim, Bcl‐2 and tRF‐31‐U5YKFN8DYDZDD (Figure S5I). The main anti‐tumour effect after interfering with tRF‐31‐U5YKFN8DYDZDD is to inhibit tumour proliferation, which is not consistent with the mechanism of 5‐Fu and can play a synergistic antitumor effect.

To verify the effect of tRF‐31‐U5YKFN8DYDZDD on angiogenesis in vivo, we detected the expression of vascular marker CD34 in the subcutaneous tumour and found that the endothelial cells with positive expression of CD34 in the tRF‐antagomir group and microvessel density (MVD) were significantly reduced compared with the control group. Compared with NC + 5‐Fu group, the number of CD34‐positive endothelial cells and MVD in tRF‐antagomir+5‐Fu group also decreased (Figure 7H). The expression of MMP2 and MMP9 also decreased in tRF‐antagomir and tRF‐antagomir+5‐Fu groups (Figure 7F). Unfortunately, at the therapeutic dose of 5‐Fu, the anti‐angiogenic effect of tRF‐antagomir alone was better than that of the combined treatment group of tRF‐antagomir and 5‐Fu (Figure 7H), indicating that 5‐Fu may affect the anti‐angiogenic effect of tRF‐antagomir.

### 3.7 Effects of tRF‐31‐U5YKFN8DYDZDD on Cell Viability and Antivascular Therapy of GC

Now, the results in vitro showed that inhibition of tRF‐31‐U5YKFN8DYDZDD increased the sensitivity to 5‐Fu, OXA and DDP, decreased cell viability and enhanced the cytotoxicity (Figure S6A,B). Bevacizumab, a humanized anti‐VEGF monoclonal antibody, is currently used as an anti‐vascular therapy in clinics for advanced GC. According to the previous research method of bevacizumab in GC [23], PBS or bevacizumab was added to the transfected cells to observe the migration. The results showed that tRF‐31‐U5YKFN8DYDZDD inhibited and bevacizumab could similarly inhibit the migration of GC, but the superposition therapy of the two had no obvious advantages (Figure S6C,D).

### 3.8 Correlation Between BMPER and Immune Cell Infiltration in GC in TCGA

Given that the in vivo experimental nude mice were immunodeficient, it was not possible to confirm the relevance of tRF‐31‐U5YKFN8DYDZDD to tumour immunotherapy. We analysed the correlation of downstream molecule BMPER with immune cell infiltration in GC tissues through the cancer genome atlas (TCGA, https://portal.gdc.cancer.gov/). It showed that BMPER in GC was correlated with the expression of multiple immunoregulatory genes such as chemokines and receptors (Figure S6E). At the same time, it was also related to the expression of multiple immune checkpoint genes (Figure S6F). We further analysed the correlation between BMPER and immune infiltration score, and found that BMPER was significantly correlated with ESTIMATScore (Comprehensive score of immunity and Matrix), Stromalscore (score of stromal cells) and Immunescore (score of immune infiltration) (Figure S6G–I). The data analysis suggests that the expression of BMPER is positively correlated with the infiltration of NK cells, mast cells, effector memory T cell, central memory T cell and CD8+ T cell macrophages, etc., but negatively related to Th17 cells and Th2 cells (Figure S6J,K). Although we found that tRF‐antagomir reduced PD‐L1 expression in xenograft tumours (Supplementary Figure S6L), the nude mouse model lacks functional T cells and cannot fully reflect immune responses. The above database information suggests that tRF‐31‐U5YKFN8DYDZDD‐BMPER may be involved in T cell‐related immune response in GC, but whether it is involved in the regulation of immunotherapy and the regulatory mechanism is worthy of further study.

## 4 Discussion

tRNA halves (tiRNAs), also known as tRNA‐derived and stress‐induced small RNAs, are induced by hypoxia, nutritional deficiency, ultraviolet radiation, heat shock, oxidative stress and viral infection [24, 25]. tRNA‐derived fragments (tRFs), which are generally considered to have four subclasses: tRF‐5, tRF‐3, endogenous tRF (i‐tRF) and tRF‐1. However, the mechanism of tRFs production is not fully understood, and it is currently accepted that it may be related to Dicer cleavage in the D‐loop or T‐loop [26]. Although the mechanisms of these two are different, all these subtypes are closely related to the occurrence and development of different tumours.

In the mechanism studies, tsRNAs were found to be involved in a variety of cellular processes, including stress response, ribosomal biogenesis, epigenetic regulation, translation repression, mRNA stability inhibition and so on [27]. On the one hand, tsRNAs can regulate tumour progression by competitively binding RNA‐binding proteins. For example, tRFs can competitively bind with YBX1 mRNA and inhibit the proliferation and invasion of breast cancer cells by stabilizing oncogene transcription [28]. On the other hand, it has been reported that tsRNAs can play an RNA silencing role similar to miRNA, and can bind to Argonaute protein to form RNA‐mediated silencing complex to achieve mRNA silencing, thus inhibiting target genes to affect the occurrence and development of tumours [29]. A 3′‐tRF, derived from tRNA‐GlyGCC, has been shown to bind to Argonaute proteins, thereby inhibiting the expression of replication protein A1 and suppressing the proliferation of B‐cell lymphomas [30]. The above two mechanisms are relatively clear at present; of course, there are some special mechanisms, such as a 3′‐tsRNA named LeuCAG which can bind to the mRNAs of ribosomal proteins to enhance effective translation regulation [31]. At present, the research on the regulation mechanism in tumour is still being deeply explored, which also provides a development space for tsRNAs to become the target of tumour therapy.

Currently, combination chemotherapy containing anti‐pyrimidine drugs is widely used as the first‐line treatment for postoperative adjuvant chemotherapy or advanced GC. However, the limitation of the therapeutic effect is a potential gap in the development of GC treatment [32]. ncRNAs can regulate gene expression, and have the specificity of targeted therapy. Compared with traditional chemotherapy drugs, these drugs have low side effects, so they have the potential to become new anti‐tumour drugs [33]. In October 2018, the United States Food and Drug Administration (FDA) approved patisiran for the treatment of polyneuropathy caused by hereditary thyroxine‐mediated amyloidosis (hATTR) in adult patients, which provided a new treatment direction for clinical [34]. From the above, it can be inferred the feasibility of developing tumour therapeutics against ncRNAs. Based on the research results in vivo and in vitro, it is suggested that the reduction of tRF‐31‐U5YKFN8DYDZDD can achieve the anti‐tumour effect of GC, and it may become a novel targeted drug for GC in the future. In nude mice, our results showed that tRF‐antagomir significantly reduced the tumour load of orthotopic transplantation, and combined with 5‐Fu enhanced the therapeutic effect. The exact mechanism is still looking forward to deeper research. The observation that tRF‐antagomir alone appeared to suppress angiogenesis more effectively than the combination with 5‐FU (Figure 7H) is intriguing but not without precedent. One plausible explanation is that 5‐FU, although cytotoxic to tumour cells, may interfere with the antagomir distribution, induce general toxicity in endothelial cells, or trigger microenvironmental changes that collectively reduce the net anti‐angiogenic effect when both agents are given concurrently. Alternatively, the endpoint MVD measurement may not capture the dynamic sequence of vascular responses. Importantly, both monotherapy and combination therapy significantly inhibited angiogenesis compared with controls. Therefore, the finding does not invalidate the therapeutic potential of tRF‐antagomir but rather underscores the importance of optimizing combination regimens. However, it is undeniable that the use of tsRNAs as RNA molecules for innovative drug research will open up a new field of biomedical science. But the nude mouse model cannot fully reflect the tumour microenvironment of actual GC patients, and further studies using xenograft models derived from patients are needed to confirm our results. In contrast to the oncogenic role of tRF‐31 identified in our study, some tRNA‐derived fragments exhibit tumour‐suppressive functions in gastric cancer. For example, tRF‐5026a inhibits GC cell proliferation via the PTEN/PI3K/AKT pathway [15]. These opposing observations underscore the context‐dependent nature of tRF biology, where the functional outcome—pro‐tumorigenic or anti‐tumorigenic—depends on the specific tRF sequence, its target genes and the cellular microenvironment. Thus, tRFs should not be generalized as uniformly oncogenic or suppressive; rather, their roles must be interpreted within the specific biological context of each cancer type and model system.

For patients with advanced or locally advanced GC, in addition to conventional chemotherapy, immunotherapy has gradually developed into an optional treatment strategy. For example, anti‐PD‐1 therapy can improve the clinical prognosis of patients with GC [35]. However, most patients with GC are resistant to PD‐1 treatment, and only a small number benefit from this immunotherapy [36]. Although we found that tRF‐antagomir reduced PD‐L1 expression in xenograft tumours, future studies using immunocompetent or humanized mouse models, as well as in vitro tumour‐immune co‐culture systems, are necessary to validate the immunomodulatory role of tRF‐31 and to explore its potential combination with immune checkpoint inhibitors. It has been reported that in the tumour microenvironment, tumour cells can recruit immunosuppressive cells, such as CD4^+^T cells, to destroy the cytotoxic function of CD8^+^T cells, thus leading to the failure of immunotherapy or drug resistance [37, 38]. CD4^+^ cells mainly include helper T cells Th1 cells, Th2 cells, Th17 cells and regulatory T cells [39]. A number of studies have shown that a variety of inducements such as surgery and trauma can lead to selective suppression of Th1 cells function and shift to Th2, resulting in cell‐mediated immunosuppression [40]. Therefore, understanding the correlation between tsRNAs and various immune cells in the tumour immune microenvironment is also the key to studying immunotherapy and its tolerability in GC. In this study, we found that BMPER, which acts downstream of tRF‐31‐U5YKFN8DYDZDD, is related to multiple immune cell infiltration in GC. However, it is only limited to the theoretical research of the database at present, and we look forward to further verification in basic and clinical trials. A better understanding of the molecular mechanism that leads to the progression and immune evasion is also a crucial part of the treatment strategy for GC.

## 5 Conclusions

1. tRF‐31‐U5YKFN8DYDZDD regulating BMPER on the malignant progression of GC through ERK and Smad signalling pathways.
2. tRF‐31‐U5YKFN8DYDZDD affected the microangiogenesis of GC cells by regulating BMPER.
3. tRF‐31‐U5YKFN8DYDZDD can increase the cytotoxicity of chemotherapy drugs and be used as a synergistic therapeutic agent.
4. tRF‐31‐U5YKFN8DYDZDD‐BMPER may be involved in T cell‐related immune response in GC.

## Author Contributions

**Yuejiao Huang:** project administration (equal), writing – original draft (equal). **Xun Li:** visualization (equal). **Yu Zhang:** methodology (equal). **Xinliang Gu:** methodology (equal). **Shiyi Qin:** software (equal), validation (equal). **Ming Zheng:** data curation (equal), supervision (equal). **Han Wang:** supervision (equal), writing – review and editing (equal). **Shaoqing Ju:** resources (equal), writing – review and editing (equal).

## Funding

This work was supported by National Natural Science Foundation of China, 82072363, 82272411. Science and Technology Support Program of Jiangsu Province, BE2023741.

## Ethics Statement

The studies involving human participants were reviewed and approved by the ethics committee of the Affiliated Hospital of Nantong University (ethical review report number: 2018‐L055). The patients/participants provided their written informed consent to participate in this study. The above animal study was performed following the Basel Declaration and the approval of the Animal Use and Care Committee of Nantong University (ethical review report number: S20221222‐006).

## Conflicts of Interest

The authors declare no conflicts of interest.

## Supporting information


**Figure S1:** Interference and overexpression efficiency of tRF‐31‐U5YKFN8DYDZDD were measured by qRT‐PCR.

**Figure S2:** Effect of overexpression of tRF‐31‐U5YKFN8DYDZDD on the biological ability of GC cells. Proliferation, migration and invasion of GC cells after overexpression of tRF‐31‐U5YKFN8DYDZDD detected by EdU (A), clone formation (B), transwell (C,D,F) and wound healing (E,F) assay.

**Figure S3:** Detection of BMPER overexpression efficiency by qRT‐PCR (A) and WB (B).

**Figure S4:** Effect of overexpression of BMPER on the biological ability of GC cells. The proliferation, migration and invasion of GC cells after overexpression of BMPER by CCK‐8 (A), clone formation (B), migration (C), invasion (D) and wound healing (F) assay. Statistical graphs of clone formation, migration, invasion (E) and wound healing (G) assays.

**Figure S5:** Effect of interference/overexpression of tRF‐31‐U5YKFN8DYDZDD on apoptosis of GC cells. Flow cytometry was used to detect the apoptosis of GC cells after interfering (A‐D,G) and overexpression (E‐F,H) of tRF‐31‐U5YKFN8DYDZDD. (I) WB was performed to detect the expression levels of Bax, Bim and Bcl‐2 in GC cells.

**Figure S6:** Effect of interfering tRF‐31‐U5YKFN8DYDZDD on treatment. (A‐B) CCK‐8 detection of the cell viability of GC cells after interference with tRF‐31‐U5YKFN8DYDZDD and addition of different chemotherapy drugs. (C‐D) Migration of GC cells after interference with tRF‐31‐U5YKFN8DYDZDD and adding Bevacizumab. (E‐K) Correlation between BMPER and immune cell infiltration in GC in TCGA. Data: RNAseq data in level 3 HTSeq‐FPKM format from the TCGA (https://portal.gdc.cancer.gov/) STAD (gastric cancer) project. (L) IHC assays show the expression of PD‐L1 in GC subcutaneous tumour tissues.

## Data Availability

All data supporting the conclusions presented in the study are included in this manuscript. Further inquiries can be directed to the corresponding authors.
